# Telomerase Inhibition Targets Clonogenic Multiple Myeloma Cells through Telomere Length-Dependent and Independent Mechanisms

**DOI:** 10.1371/journal.pone.0012487

**Published:** 2010-09-01

**Authors:** Sarah K. Brennan, Qiuju Wang, Robert Tressler, Calvin Harley, Ning Go, Ekaterina Bassett, Carol Ann Huff, Richard J. Jones, William Matsui

**Affiliations:** 1 Department of Oncology, The Sidney Kimmel Comprehensive Cancer Center, The Johns Hopkins University School of Medicine, Baltimore, Maryland, United States of America; 2 Geron Corporation, Menlo Park, California, United States of America; Garvan Institute of Medical Research, Australia

## Abstract

**Background:**

Plasma cells constitute the majority of tumor cells in multiple myeloma (MM) but lack the potential for sustained clonogenic growth. In contrast, clonotypic B cells can engraft and recapitulate disease in immunodeficient mice suggesting they serve as the MM cancer stem cell (CSC). These tumor initiating B cells also share functional features with normal stem cells such as drug resistance and self-renewal potential. Therefore, the cellular processes that regulate normal stem cells may serve as therapeutic targets in MM. Telomerase activity is required for the maintenance of normal adult stem cells, and we examined the activity of the telomerase inhibitor imetelstat against MM CSC. Moreover, we carried out both long and short-term inhibition studies to examine telomere length-dependent and independent activities.

**Methodology/Principal Findings:**

Human MM CSC were isolated from cell lines and primary clinical specimens and treated with imetelstat, a specific inhibitor of the reverse transcriptase activity of telomerase. Two weeks of exposure to imetelstat resulted in a significant reduction in telomere length and the inhibition of clonogenic MM growth both *in vitro* and *in vivo*. In addition to these relatively long-term effects, 72 hours of imetelstat treatment inhibited clonogenic growth that was associated with MM CSC differentiation based on expression of the plasma cell antigen CD138 and the stem cell marker aldehyde dehydrogenase. Short-term treatment of MM CSC also decreased the expression of genes typically expressed by stem cells (*OCT3/4*, *SOX2*, *NANOG,* and *BMI1*) as revealed by quantitative real-time PCR.

**Conclusions:**

Telomerase activity regulates the clonogenic growth of MM CSC. Moreover, reductions in MM growth following both long and short-term telomerase inhibition suggest that it impacts CSC through telomere length-dependent and independent mechanisms.

## Introduction

Multiple myeloma (MM) is characterized by the clonal expansion of malignant plasma cells that results in anemia, renal insufficiency, and bone disease. The introduction of several novel agents has steadily improved therapeutic responses and clinical outcomes in MM [1]. However, most patients eventually relapse and die as a consequence of their disease despite these improvements. Tumor re-growth following initial reductions in disease burden suggests that tumor cells capable of clonogenic growth are relatively drug resistant, and in several human cancers these functional properties have been attributed to cancer stem cells (CSC). We previously found that MM cells responsible for producing symptomatic disease in immunodeficient mice phenotypically resemble memory B cells rather than mature plasma cells [2], [3]. Furthermore, these clonotypic B cells exhibit several attributes that characterize normal stem cells including self-renewal during serial transplantation and multi-drug resistance. These shared functional properties suggest that specific cellular processes regulating normal stem cells may be active within CSC and serve as potential therapeutic targets.

Telomerase is a ribonuclear protein complex minimally consisting of protein (TERT) and RNA (TERC) components that act as a reverse transcriptase to extend telomeres at the ends of linear chromosomes [4], [5]. In the absence of telomerase activity (TA), telomeres undergo progressive shortening during successive replication cycles, eventually reaching critically short lengths that result in cellular senescence or apoptosis. Telomerase activation is a common feature of nearly all human cancers and is believed to support continued cell replication [6], [7]. In MM, the malignant plasma cells from virtually all patients express activated telomerase, and higher TA correlates with decreased overall survival [8], [9]. Several studies have also demonstrated that the inhibition of TA in MM plasma cells results in progressive telomere shortening and growth inhibition both *in vitro* and *in vivo* [10]–[21].

In normal adult tissues, telomerase is most active in long-lived stem cells and progenitors, and then progressively decreases in more differentiated progeny [22]–[24]. TA is also required for the maintenance of normal stem cells, and in patients with dyskeratosis congenita (DKC) inactivating mutations in *TERT* and *TERC* may lead to the loss of normal hematopoietic stem cells and aplastic anemia [25]. Thus, we studied the role of telomerase in MM CSC by examining the effects of imetelstat, a specific competitive inhibitor of the telomerase reverse transcriptase activity [26]. We found that TA inhibition in MM CSC for two weeks or longer progressively reduced telomere length and decreased both colony formation *in vitro* and the engraftment of immunodeficient mice. In addition, short-term imetelstat treatment of MM CSC for 72 hours resulted in clonogenic growth inhibition associated with the induction of differentiation, but not telomere shortening. Therefore, TA inhibition may impact MM CSC both by decreasing telomere length and modulating self-renewal programs.

## Results

### Imetelstat inhibits TA in MM plasma cells and CSC

**Figure 1:**
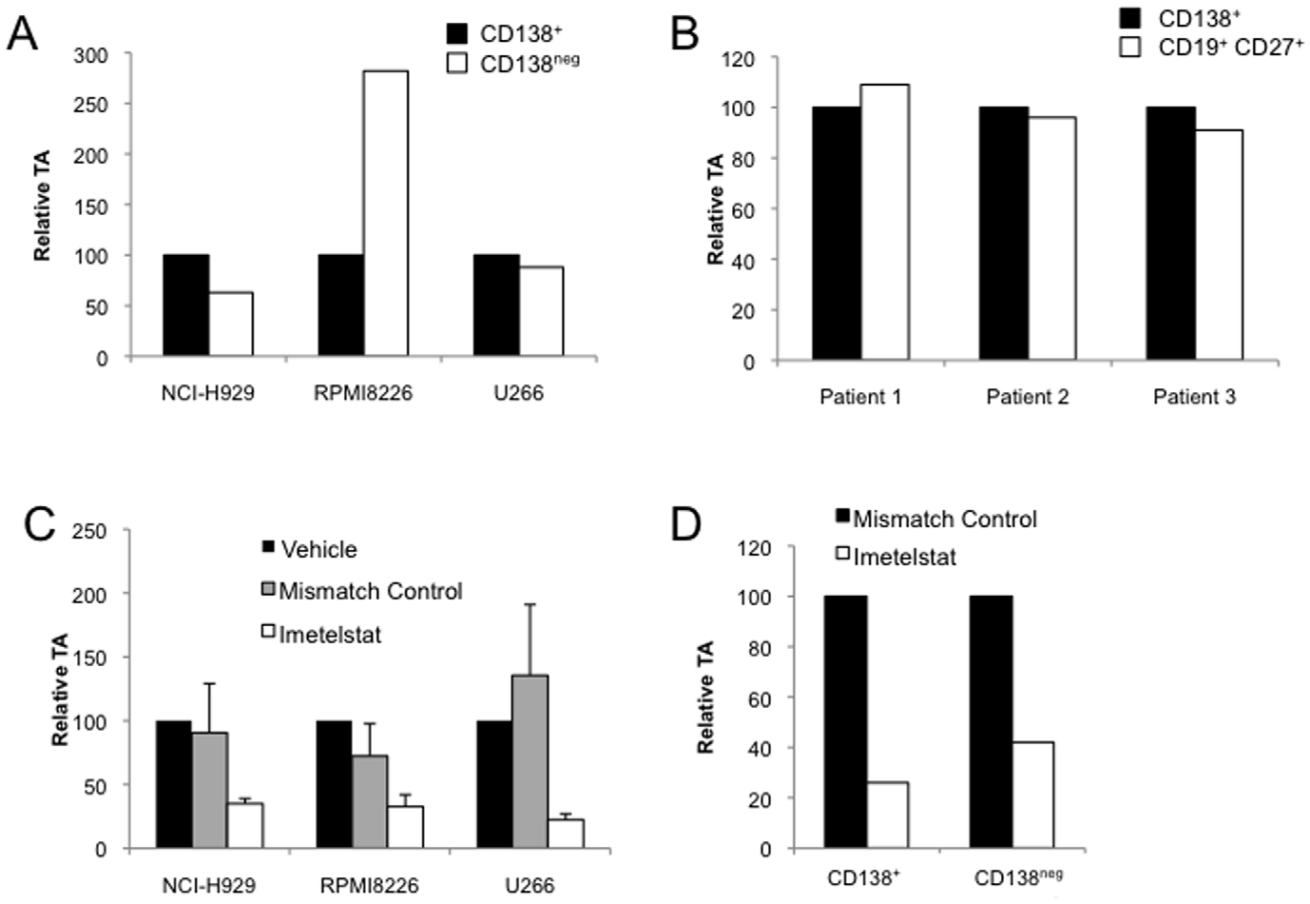
Telomerase is active and can be inhibited in MM CD138^+^ and CD138^neg^ cells. (A) Relative telomerase activity in CD138^+^ plasma cells and CD138^neg^ CSC isolated from MM cell lines. (B) Relative telomerase activity in CD138^+^ plasma cells and CD19^+^CD27^+^ CSC isolated from clinical MM bone marrow samples. (C) Relative telomerase activity in bulk MM cell lines following 72 hours of treatment with vehicle, mismatch control oligonucleotide, or imetelstat (1 uM). (D) Relative telomerase activity in CD138^+^ and CD138^neg^ NCI-H929 cells after 72 hours of treatment with mismatch control oligonucleotide or imetelstat (1 uM).

MM plasma cells express the characteristic surface antigen CD138, but the self-renewing cells responsible for tumor initiation in immunodeficient mice phenotypically resemble memory B cells and are CD138^neg^ [2]. We initially examined relative TA within CSC and bulk tumor cells and isolated CD138^neg^ precursors and mature CD138^+^ plasma cells from pre-sorted MM cell lines. In the RPMI8226 cell line, CD138^neg^ CSC displayed greater TA than CD138^+^ plasma cells, while this pattern was reversed in NCI-H929 cells (Figure 1A). In the U266 cell line, TA was similar between the two cell compartments. We also studied primary clinical specimen from three distinct patients and found that the levels of TA were similar in CD138^neg^CD19^+^CD27^+^ precursors and CD138^+^ plasma cells (Figure 1B). Taken together, these results demonstrate that telomerase is active in both MM CSC and plasma cells, but relative levels within each cell compartment may vary within individual cell lines and patient specimens.

Several approaches have been developed to target telomerase including antisense RNA directed against *TERT*, small molecules that stabilize G-quadruplexes, and oligonucleotides acting as direct inhibitors of the reverse transcriptase activity [27]–[31]. Imetelstat is a synthetic lipid-conjugated 13-base oligonucleotide N3′ P5′-thio-phosphoramidate complementary to the template region of telomerase RNA (*TERC*) that acts as a competitive enzyme inhibitor by binding and blocking the active site of the enzyme [10], [11], [14]. This highly specific inhibitor of TA has been clinically studied in patients with MM, chronic lymphocytic leukemia, non-small cell lung cancer, and breast cancer [26], [32]. We studied the ability of imetelstat to inhibit TA in MM and treated RPMI8226, NCI-H929, and U266 cells for 48 hours. Using the TRAP assay, we detected at least a 60% reduction in TA in cells treated with imetelstat compared to a mismatched control oligonucleotide (Figure 1C). Similar degrees of telomerase inhibition have been reported in MM and glioblastoma cells [11], [33]. We also examined telomerase inhibition in CD138^neg^ precursors and CD138^+^ plasma cells isolated from the NCI-H929 cell line and found that imetelstat inhibited TA by 58% and 74% in each of these cell populations, respectively (Figure 1D).

### Imetelstat reduces telomere length and limits the clonogenic growth of CD138^neg^ MM CSC

**Figure 2:**
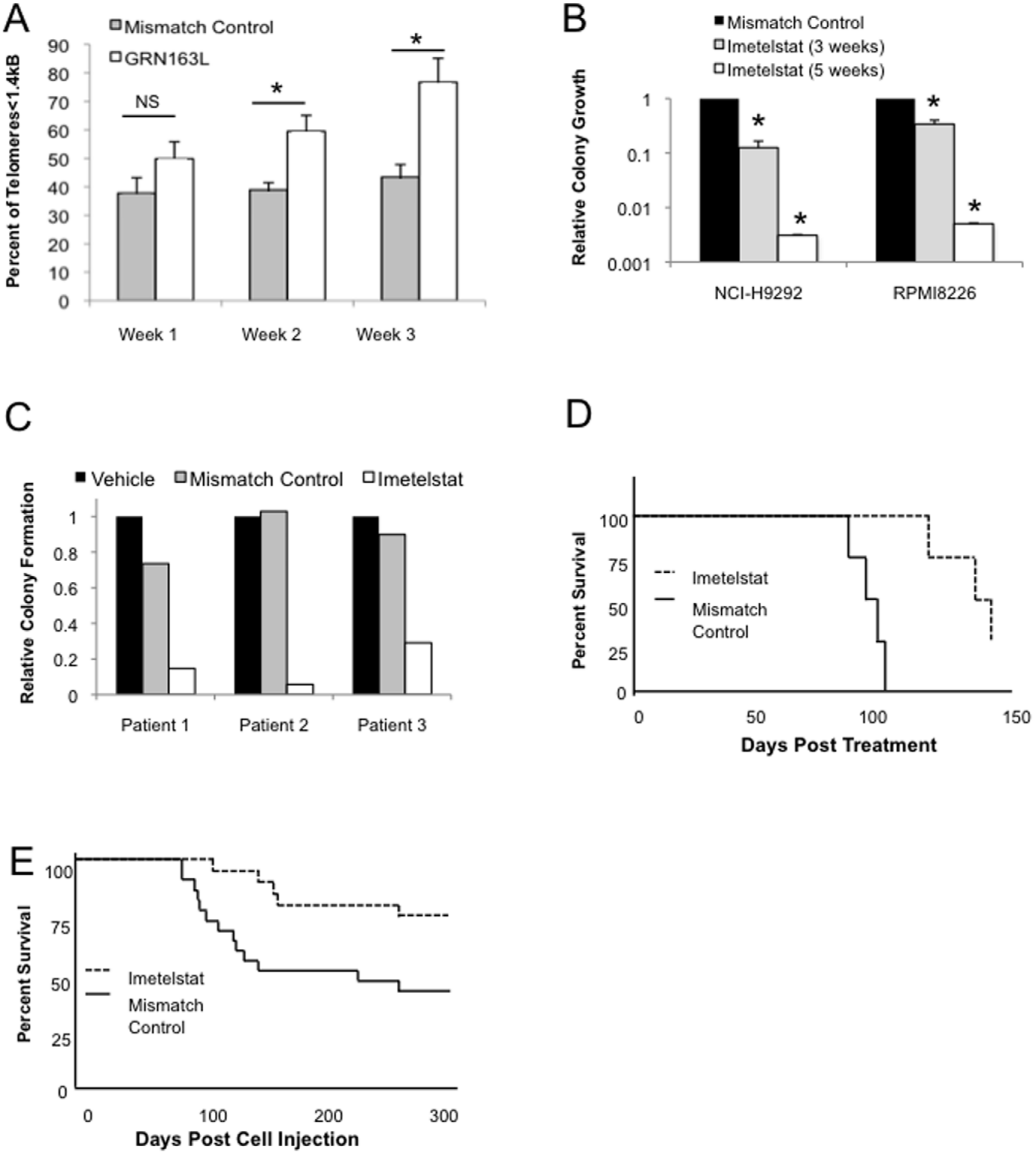
Prolonged telomerase inhibition decreases MM CSC telomere length and clonogenic potential. (A) Percentage of telomeres less than 1.4 kb as determined by STELA after 1, 2, or 3 weeks with mismatch control oligonucleotide or imetelstat (*p-value <0.05 and NS; not significant). (B) Relative clonogenic growth of CD138^neg^ NCI-H929 and RPMI8226 cells after 3 and 5 weeks of treatment with mismatch control oligonucleotide or imetelstat (1 uM). (C) Relative clonogenic recovery of CD138^neg^ cells isolated from primary clinical specimens following 3 weeks of treatment with vehicle, mismatch control oligonucleotide or imetelstat (1 uM). (D) Survival of NOD/SCID mice injected with NCI-H929 cells then treated with imetelstat (solid line) or mismatch control oligonucleotide (dashed line) for two weeks *in vivo* (p<0.01, n = 8,). (E) Survival of NOD/SCID mice after injection with NCI-H929 cells following in vitro treatment with imetelstat (solid line) or mismatch control oligonucleotide (dashed line) for two weeks (*P*<0.001, n = 20 per group).

Several reports have demonstrated that telomerase inhibition in CD138^+^ MM plasma cells can reduce telomere length to a critical threshold resulting in senescence and/or apoptosis[10]–[14], [34]. We examined the impact of imetelstat on MM CSC telomere length and continuously treated pre-sorted CD138^neg^ cells isolated from the NCI-H929 cell line. By two weeks, cells treated with imetelstat displayed a significantly increased proportion (p<0.01, Figure 2A) of shortened telomeres (<1.4 kb) using the single telomere length assay (STELA)[35]. In order to examine the effects of telomerase inhibition and progressive telomere shortening on the clonogenic growth of MM CSC, CD138^neg^ cells were isolated from the RPMI8226 and NCI-H929 cell lines, continuously treated with imetelstat, and plated weekly in methylcellulose. Colony formation was progressively inhibited with a 5-fold reduction at three weeks and greater than 100-fold reduction after five weeks of treatment (Figure 2B). We also examined primary clinical MM specimens and treated pre-sorted CD138^neg^ bone marrow cells with imetelstat. Compared to the control oligonucleotide, MM clonogenic growth was significantly inhibited by approximately 5-fold after 3 weeks of imetelstat treatment compared to the mismatch control group (*P*<0.01, Figure 2C).

We also examined the effects of imetelstat on a MM growth *in vivo*. Previous studies demonstrated that imetelstat reduced the growth of subcutaneous tumors in SCID mice [11], [14]. In order to examine the effects of imetelstat on systemic MM growth, NOD/SCID mice were injected with NCI-H929 cells then treated with imetelstat two weeks later. Treatment with imetelstat given three times a week for a total of two weeks significantly extended survival compared to the control mismatch oligonucleotide (p<0.01, Figure 2D). Since this study could not distinguish between the inhibition of bulk tumor growth or the self-renewal capacity of CSC, we next examined the effect of imetelstat on MM engraftment, a surrogate measure of self-renewal potential. We treated NCI-H929 cells with imetelstat or mismatch control for two weeks *in vitro* and subsequently injected them intravenously into NOD/SCID mice. Pre-treatment of MM cells with imetelstat significantly reduced the proportion and survival of mice engrafting with MM compared to control oligonucleotide treated cells (p<0.001, Figure 2E). Therefore, telomerase inhibition by imetelstat significantly reduces MM clonogenic potential both *in vitro* and *in vivo* in parallel with its effects on telomere length.

### Short-term telomerase inhibition promotes MM differentiation and depletes CSC activity

**Figure 3:**
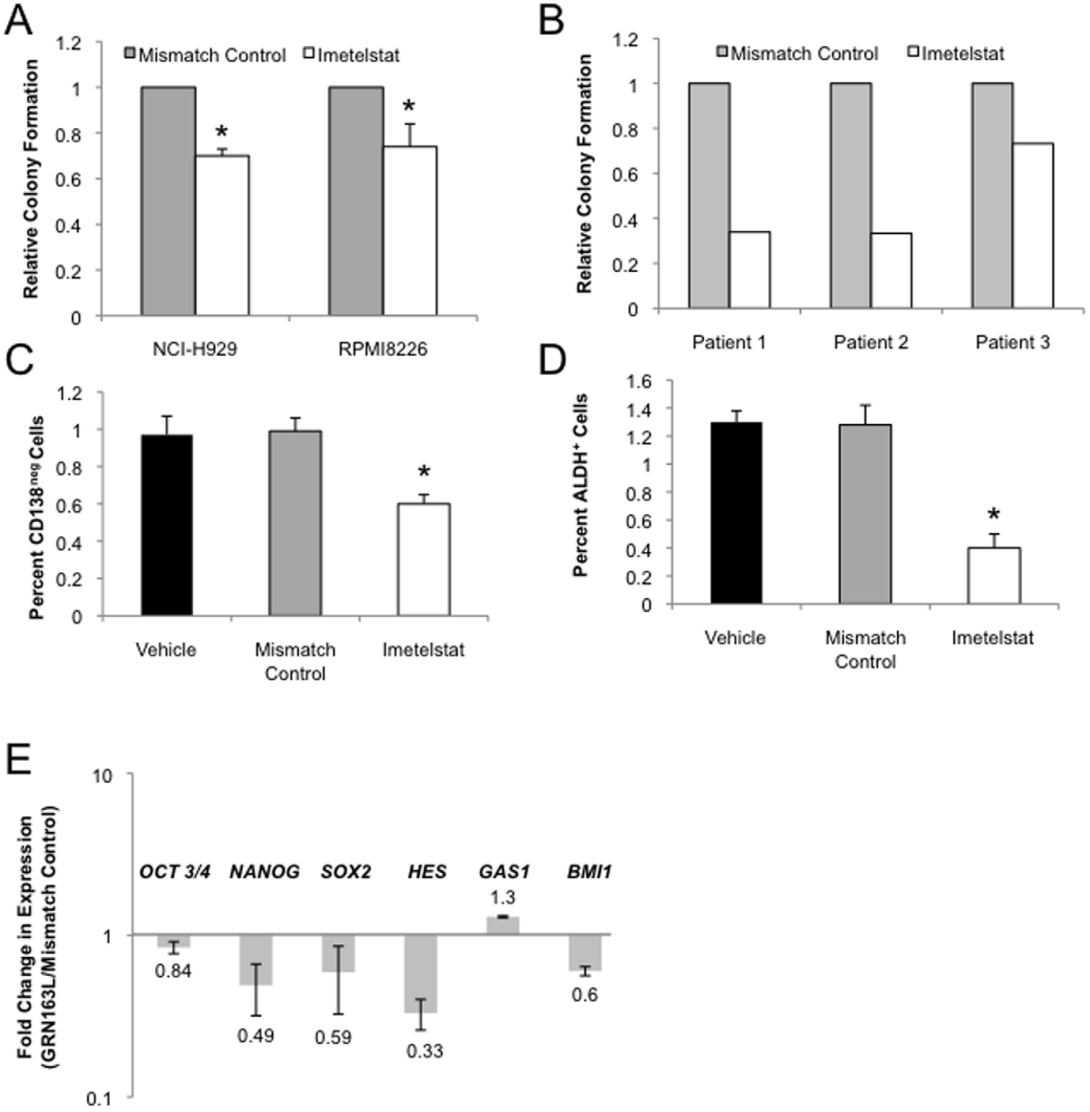
Short-term TA inhibition reduces MM CSC activity and promotes differentiation. (A) Relative clonogenic recovery of CD138^neg^ NCI-H929, RPMI8226, and U266 cells after treatment with vehicle, mismatch control oligonucleotide or imetelstat for 72 hrs (* p-value<0.01). (B) Relative clonogenic recovery of CD138^neg^ cells isolated from primary MM specimens following 72 hrs of treatment with vehicle, mismatch control oligonucleotide or imetelstat (1 uM). (C) Percentage of CD138^neg^ NCI-H929 cells following treatment with imetelstat (1 uM) for 72 hrs (* p-value<0.01). (D) Percentage of ALDH^+^ NCI-H929 cells following treatment with imetelstat (1 uM) for 72 hrs (* p-value<0.01). (E) Expression of *OCT3/4, SOX2, NANOG, HES, GAS1* and *BMI1* by CD138^neg^ NCI-H929 cells after 72 hrs of treatment with imetelstat or mismatched control oligonucleotide (1 uM). Data represent fold change in expression relative to mismatch control treated values.

During the long-term treatment studies, we found that the clonogenic growth of pre-sorted CD138^neg^ RPMI8226, NCI-H929, and U266 MM CSC was significantly inhibited by 11–30% following just 72 hours of imetelstat exposure (p<0.01, Figure 3A), although telomere length was not significantly affected in NCI-H929 cells after 1 week of treatment (p>0.05, Figure 2A). Moreover, this effect was not mediated by the induction of cell death as we found no difference in the proportion of apoptotic cells following 72 hours of exposure to imetelstat (data not shown). Similarly, we found that short-term treatment of pre-sorted CD138^neg^ bone marrow cells from primary clinical specimens with imetelstat significantly inhibited MM colony formation by 19-29% compared to mismatch control treated samples (p<0.05, Figure 3B). These data suggest that short-term imetelstat treatment in MM impacts the functional capacity of MM CSC independent of its effects on telomere length.

Several recent reports have suggested that telomerase may regulate the differentiation and self-renewal of normal stem cells independent of its effects on telomere length [33], [36]. Therefore, we examined the impact of short-term imetelstat treatment on the differentiation of MM CSC. Treating presorted CD138^neg^ with imetelstat, control oligo or vehicle, resulted in increased expression of CD138 after just 72 hours without a significant difference between the groups (Data not shown). To specifically evaluate the effects of imetelstat on this process we evaluated the proportion of CD138^neg^ cells in unsorted bulk cultures after 72 hours. We found that the proportion of CD138^neg^ cells was decreased by approximately 40% after short-term imetelstat treatment of unsorted H929 cells (p<0.01, Figure 3C). MM CSC also express relatively higher levels of ALDH activity compared to CD138^+^ plasma cells [2], and short-term imetelstat treatment significantly decreased the ALDH^+^ population from 1.3% to less than 0.4% (p<0.01, Figure 3D). These data suggest that short term TA inhibition depletes the population of MM CSC by inducing their terminal differentiation.

We hypothesized that telomerase may globally influence pathways that regulate CSC cell-fate decisions and self-renewal. Within embryonic stem cells, specific transcriptional regulators, such as *OCT3/4, NANOG,* and *SOX2* are critical for regulating self-renewal and pluripotency [37]–[39]. Moreover, developmental signaling pathways, such as Notch and Hedgehog, as well as the polycomb gene *BMI1* are thought to regulate both normal and cancer stem cells [40]–[43]. We examined the expression of *OCT3/4, NANOG, SOX2* and *BMI1* as well as the Hedgehog and Notch target genes, *GAS1* and *HES1* in NCI-H929 cells and found that all were detectable in the CD138^neg^ CSC, but not the bulk CD138^+^ plasma cells (data not shown). Following treatment of CD138^neg^ NCI-H929 CSC with imetelstat for 72 hours, the expression of five out of six of these stem cell genes was inhibited compared to mismatch control treated cells (Figure 3E). These results suggest that short-term telomerase inhibition may directly impact stem cell programs that regulate CSC self-renewal.

## Discussion

Highly clonogenic CSC have been identified in several human malignancies, including MM, and their combined resistance to chemotherapy and enhanced growth potential suggests that they may be responsible for disease relapse and progression [2], [3]. Thus, the development of therapeutic agents that effectively target CSC may improve long-term outcomes, such as progression-free and overall survival. Few clinically applicable strategies have been identified to date, but the cellular processes that regulate normal stem cells, such as developmental signaling pathways, have emerged as potential targets [44].

Increasing evidence suggests that telomerase is essential for normal stem cell function. For example, TA is highest within stem cells in rapidly proliferating tissues such as the bone marrow and gut, and the longest telomeres within the skin are found with stem cells [7], [45]–[48]. The requirement for telomerase by normal stem cells is illustrated by DKC in which inactivating mutations in *TERT* or *TERC* result in degenerative failure of multiple organs including the bone marrow from hematopoietic stem cell exhaustion [25], [49], [50]. The anticipatory nature of DKC in which successive generations experience disease manifestations at younger ages suggests that telomere length rather than TA is the primary factor that regulates normal stem cell function [5]. Similarly, *Tert^null/null^* or *Terc^null/null^* mice lacking TA do not demonstrate degenerative phenotypes until they acquire critically short telomeres through successive rounds of interbreeding [51], [52]. In MM, several reports have demonstrated that TA must be inhibited for at least 2 to 3 weeks to reduce telomere length in MM plasma cells and affect their growth and survival [11]–[13], [53]. We examined CD138^neg^ MM CSC and also found that telomere shortening following 2 weeks of exposure to imetelstat was associated with decreased clonogenic growth both *in vitro* and *in vivo.* Similar findings have been reported in glioblastoma [33]; thus, telomerase inhibition may target CSC across multiple tumor types.

The efficacy of long-term telomerase inhibition suggests that imetelstat modulates MM CSC growth and self-renewal primarily by modulating telomere length. But we also found that short-term drug exposure significantly inhibits clonogenic MM growth associated with CSC differentiation. Therefore, telomerase inhibition may impact CSC function through both telomere length-dependent and independent mechanisms. In a similar fashion, short-term telomerase inhibition has been found to induce the differentiation of medulloblastoma and melanoma cells as well as sensitize glioblastoma CSC to cytotoxic chemotherapy [33], [54]–[56]. In normal stem cells several studies have also suggested that telomerase may impact self-renewal and differentiation programs independent of effects on telomere length [57]–[59], but these results contrast those in which both *Tert^null/null^* and *Terc^null/null^* mice display no abnormalities until telomeres are shortened to critical lengths [51], [52]. Interestingly, hematopoietic stem cells isolated from early generation *Terc^null/null^* mice with normal peripheral blood counts are functionally impaired when exposed to the stress of bone marrow transplantation [60], [61]. Therefore, normal HSC may have different requirements for intact telomerase activity depending on whether they involved in homeostatic or stress hematopoiesis. In cancer, telomerase activity exclusive of its effects on telomere length may be particularly evident since CSC may display a high degree of metabolic stress as demonstrated in acute leukemia [62].

We also found that short-term telomerase inhibition results in decreased expression of genes, such as *OCT3/4*, *NANOG, SOX2,* and *BMI1*, that regulate normal and cancer stem cells. Similarly, TERT has also been found to directly activate epithelial stem cells by inducing a Myc- and Wnt-related transcriptional program [36]. Furthermore, TERT is directly associated with the promoters of Wnt target genes [63]. These findings suggest that telomerase may interact with essential pathways during stem cell-fate decisions and that modulation of these other pathways may further enhance the anti-tumor activity of telomerase inhibitors.

## Materials and Methods

### Patient samples, cell lines and cell culture

Bone marrow samples were obtained from patients with active MM granting signed consent for sample acquisition using a protocol and consent forms approved by the Johns Hopkins Medical Institutes Review Board. All patients were newly diagnosed and included those with Stage II (n = 2) and Stage 3 (n = 4) disease according to International Staging System (ISS) criteria [64]. Mononuclear cells were isolated by density centrifugation (Ficoll-Paque, Pharmacia, Piscataway, NJ) and depleted of CD34^+^ hematopoietic progenitors using CD34 magnetic microbeads (Miltenyi Biotech, Auburn, CA). Plasma cells were further isolated by positive magnetic selection using CD138 selection beads (Miltenyi Biotech). CD138^neg^CD19^+^CD27^+^ MM CSC were isolated by FACS (see below). Isolated cells were also assessed for clonotypic immunoglobulin light chain expression by flow cytometry and demonstrated >90% isotype restriction matching the malignant plasma cells.

The human MM cells lines NCI-H929, RPMI8226 and U266 were obtained from the American Type Tissue Collection (Manassas, VA). All cells were cultured in complete media consisting of RPMI 1640, 2 mM L-glutamine, 50 U/mL penicillin, 50 µg/mL streptomycin, and 10% fetal bovine serum (FBS). For drug treatment studies cells were treated with 1 µM imetelstat, GRN163L (5′-palm-TAGGGTTAGACAA-3′) or 1 µM mismatch control oligonucleotide, GRN140833 (5′-palm-TAGGTGTAAGCAA-3′) (Geron Corporation, Menlo Park, CA) prepared in phosphate buffered saline.

For the analysis of *in vitro* clonogenic growth treated cells were washed free of drug and plated at a density of 1,000 cells/mL for cell lines or 200,000 cells/mL for CD138^neg^CD34^neg^ bone marrow mononuclear cells in 1.2% methylcellulose, 30% FBS, 1% bovine serum albumin (BSA), 10^−4^ M 2-mercaptoethanol, and 2 mM L-glutamine. For clinical specimens, the methylcellulose cultures also contained 10% lymphocyte conditioned media as a source of growth factors [2]. Samples were plated in triplicate onto 35 mm^2^ tissue culture dishes and incubated at 37°C and 5% CO_2_. Colonies consisting of more than 40 cells were scored between 14 and 21 days with an inverted microscope. Serial replating was performed by collecting methylcellulose and washing cells in complete media then resuspending a fixed proportion (1/10–1/1,000) of the cells in 1 ml of methylcellulose as described above.

### Telomerase activity and telomere length quantification

Telomerase activity was quantified using a real-time PCR based quantitative telomerase detection kit according to manufacturer's instructions (Allied Biotech Inc., Ijamsville, MD). Equal cell numbers (1×10^5^–10^6^ cells) were used for each experiment and relative telomerase activity was calculated based on the value of 2 ∧ difference in C_T_ value. XpYp single telomere length assay (STELA) was performed using the methods described by Baird et al.[35] Telomere signals were detected using TeloTAGGG Telomere Length Assay kit (Roche, San Francisco, CA). Four separate PCR reactions (1–2 ng genomic DNA per reaction) were set up for each sample and run in separate lanes on agarose gels. Total number of telomere bands from the lanes for each sample were pooled and calculated. Telomere shortening was quantified by determining the percentage of telomere bands less than 1.4 kb to the total number of bands in the sample.

### Fluorescence-activated cell sorting and flow cytometry

For antigen expression evaluation and cell sorting, anti-human CD138-fluourescein isothyocyanate (FITC), CD19-FITC, and CD27-allophycocyanin (APC) antibodies were used (BD Pharmingen, San Diego, CA). Aldehyde dehydrogenase (ALDH) activity was detected using the Aldefluor reagent (Stem Cell Technologies, Vancouver, Canada) according to manufacturer's instructions. Control staining reactions consisted of isotypic antibodies or addition of the specific ALDH inhibitor diethylaminobenzaldehyde (DEAB). CD138^+^ and CD138^neg^ cells were isolated from human MM cell lines by staining with anti-CD138 as well as 2 µg/ml propidium iodide (PI, BD Biosciences, San Jose, CA). Cells were initially gated on a FACSAria cell sorter (BD Biosciences) by forward and orthogonal light scatter, then analysed to collect CD138^neg^PI^neg^ and CD138^+^PI^neg^ cells. For clinical specimens, MM CSC were isolated by staining peripheral blood mononuclear cells with anti-CD19, anti-CD27, and PI. Cells were initially gated to eliminate cellular aggregates and necrotic material by forward and orthogonal light scatter and PI exclusion, then analyzed for CD19 and CD27 expression. Apoptosis analysis was performed using Annexin V (BD Biosciences) and PI staining. Briefly, cells were incubated with Annexin V-FITC in Annexin binding buffer followed by staining with 5 µg/ml PI and analysis by flow cytometry. Annexin^neg^PI^neg^ cells were considered viable and compared across treatment groups. Cell surface antigen or ALDH expression was examined by counterstaining cells with 2 µg/ml PI followed by analysis of cells initially gated to eliminate cellular aggregates and necrotic material by forward and orthogonal light scatter and PI exclusion quantification. Cells were analyzed using either a FACSCalibur flow cytometer (BD Biosciences) or FACSAria cell sorter.

### Transplantation of MM cells into NOD/SCID mice

All animal studies were approved by the Johns Hopkins Medical Institutes Animal Care Committee. Nonobese diabetic/severe combined immunodeficiency (NOD/SCID) mice were bred and maintained in the Johns Hopkins animal core facility. NCI-H929 cells (1×10^6^–10^7^ cells/animal) were injected into the tail vein of 6–10 week old mice. For *in vivo* treatment experiments, imetelstat or mismatch control oligonucleotide (30 mg/kg) was injected intraperitoneally three times a week for two weeks. Mice were observed daily and sacrificed following the development of symptoms. MM engraftment was confirmed by detecting human κ immunoglobulin light chain in mouse sera using ELISA (Bethyl Labs, Montgomery, TX) and human CD138^+^ plasma cells in the mouse bone marrow by flow cytometry.

### Reverse transcriptase PCR

RNA was isolated using RNAqueous (Ambion, Austin, TX), and cDNA made using Superscript II (Invitrogen) with random hexamers according to the manufacture's protocol. For quantitative RT-PCR Taqman real-time primer/probes sets (Applied Biosystems, Foster City, CA) were used to detect cDNA for *GAS1* (Hs00266715_s1), *NANOG* (Hs0238700_g1), *SOX2* (Hs01053049_s1), *HES* (Hs00707120_s1), *OCT3/4* (Hs00999652_g1) and *BMI1* (Hs00180411_m1) and *β-actin* (4352935E). PCR was performed using TaqMan Universal Master Mix (4304437, ABI) on an I-Cycler Real-Time PCR machine (BioRad, Hercules, CA). Expression levels were normalized to *β-actin* and compared with the ΔΔC_T_ method.

### Statistical analysis

Results are presented as the mean ± SEM. Comparisons between groups were performed using a 2-tailed, paired Student *t* test. Kaplan-Meier analysis was carried out using a log rank test. *P* values <0.05 were considered significant.

## References

[1] Laubach JP, Mahindra A, Mitsiades CS, Schlossman RL, Munshi NC (2009). The use of novel agents in the treatment of relapsed and refractory multiple myeloma.. Leukemia.

[2] Matsui W, Huff CA, Wang Q, Malehorn MT, Barber J (2004). Characterization of clonogenic multiple myeloma cells.. Blood.

[3] Matsui W, Wang Q, Barber JP, Brennan S, Smith BD (2008). Clonogenic multiple myeloma progenitors, stem cell properties, and drug resistance.. Cancer research.

[4] Yu GL, Bradley JD, Attardi LD, Blackburn EH (1990). In vivo alteration of telomere sequences and senescence caused by mutated Tetrahymena telomerase RNAs.. Nature.

[5] Greider CW, Blackburn EH (1985). Identification of a specific telomere terminal transferase activity in Tetrahymena extracts.. Cell.

[6] Shay JW, Bacchetti S (1997). A survey of telomerase activity in human cancer.. European journal of cancer (Oxford, England: 1990).

[7] Kim NW, Piatyszek MA, Prowse KR, Harley CB, West MD (1994). Specific association of human telomerase activity with immortal cells and cancer.. Science.

[8] Shiratsuchi M, Muta K, Abe Y, Motomura S, Taguchi F (2002). Clinical significance of telomerase activity in multiple myeloma.. Cancer genetics and cytogenetics.

[9] Wu KD, Orme LM, Shaughnessy J, Jacobson J, Barlogie B (2003). Telomerase and telomere length in multiple myeloma: correlations with disease heterogeneity, cytogenetic status, and overall survival.. Blood.

[10] Akiyama M, Hideshima T, Shammas MA, Hayashi T, Hamasaki M (2003). Effects of oligonucleotide N3′–>P5′ thio-phosphoramidate (GRN163) targeting telomerase RNA in human multiple myeloma cells.. Cancer research.

[11] Shammas MA, Koley H, Bertheau RC, Neri P, Fulciniti M (2008). Telomerase inhibitor GRN163L inhibits myeloma cell growth in vitro and in vivo.. Leukemia: official journal of the Leukemia Society of America, Leukemia Research Fund, UK.

[12] Shammas MA, Shmookler Reis RJ, Akiyama M, Koley H, Chauhan D (2003). Telomerase inhibition and cell growth arrest by G-quadruplex interactive agent in multiple myeloma.. Molecular cancer therapeutics.

[13] Shammas MA, Shmookler Reis RJ, Li C, Koley H, Hurley LH (2004). Telomerase inhibition and cell growth arrest after telomestatin treatment in multiple myeloma.. Clinical cancer research: an official journal of the American Association for Cancer Research.

[14] Wang ES, Wu K, Chin AC, Chen-Kiang S, Pongracz K (2004). Telomerase inhibition with an oligonucleotide telomerase template antagonist: in vitro and in vivo studies in multiple myeloma and lymphoma.. Blood.

[15] Guo C, Geverd D, Liao R, Hamad N, Counter CM (2001). Inhibition of telomerase is related to the life span and tumorigenicity of human prostate cancer cells.. The Journal of urology.

[16] Miura N, Horikawa I, Nishimoto A, Ohmura H, Ito H (1997). Progressive telomere shortening and telomerase reactivation during hepatocellular carcinogenesis.. Cancer genetics and cytogenetics.

[17] Hashizume R, Ozawa T, Gryaznov SM, Bollen AW, Lamborn KR (2008). New therapeutic approach for brain tumors: Intranasal delivery of telomerase inhibitor GRN163.. Neuro-oncology.

[18] Kraemer K, Fuessel S, Meye A (2007). Telomerase inhibition by synthetic nucleic acids and chemosensitization in human bladder cancer cell lines.. Methods in molecular biology (Clifton, NJ).

[19] Engelhardt M, Mackenzie K, Drullinsky P, Silver RT, Moore MA (2000). Telomerase activity and telomere length in acute and chronic leukemia, pre- and post-ex vivo culture.. Cancer research.

[20] Hochreiter AE, Xiao H, Goldblatt EM, Gryaznov SM, Miller KD (2006). Telomerase template antagonist GRN163L disrupts telomere maintenance, tumor growth, and metastasis of breast cancer.. Clinical cancer research: an official journal of the American Association for Cancer Research.

[21] Dikmen ZG, Gellert GC, Jackson S, Gryaznov S, Tressler R (2005). In vivo inhibition of lung cancer by GRN163L: a novel human telomerase inhibitor..

[22] Hiyama K, Hirai Y, Kyoizumi S, Akiyama M, Hiyama E (1995). Activation of telomerase in human lymphocytes and hematopoietic progenitor cells.. J Immunol.

[23] Harle-Bachor C, Boukamp P (1996). Telomerase activity in the regenerative basal layer of the epidermis inhuman skin and in immortal and carcinoma-derived skin keratinocytes.. Proc Natl Acad Sci U S A.

[24] Yui J, Chiu CP, Lansdorp PM (1998). Telomerase activity in candidate stem cells from fetal liver and adult bone marrow.. Blood.

[25] Armanios M, Chen JL, Chang YP, Brodsky RA, Hawkins A (2005). Haploinsufficiency of telomerase reverse transcriptase leads to anticipation in autosomal dominant dyskeratosis congenita.. Proc Natl Acad Sci U S A.

[26] Herbert BS, Pongracz K, Shay JW, Gryaznov SM (2002). Oligonucleotide N3′–>P5′ phosphoramidates as efficient telomerase inhibitors.. Oncogene.

[27] Feng J, Funk WD, Wang SS, Weinrich SL, Avilion AA (1995). The RNA component of human telomerase.. Science.

[28] Galderisi U, Cascino A, Giordano A (1999). Antisense oligonucleotides as therapeutic agents.. J Cell Physiol.

[29] Yokoyama Y, Takahashi Y, Shinohara A, Lian Z, Wan X (1998). Attenuation of telomerase activity by a hammerhead ribozyme targeting the template region of telomerase RNA in endometrial carcinoma cells.. Cancer Res.

[30] Zhang X, Mar V, Zhou W, Harrington L, Robinson MO (1999). Telomere shortening and apoptosis in telomerase-inhibited human tumor cells.. Genes Dev.

[31] Strahl C, Blackburn EH (1996). Effects of reverse transcriptase inhibitors on telomere length and telomerase activity in two immortalized human cell lines.. Mol Cell Biol.

[32] Brower V Telomerase-based therapies emerging slowly.. J Natl Cancer Inst.

[33] Marian CO, Cho SK, McEllin BM, Maher EA, Hatanpaa KJ The telomerase antagonist, imetelstat, efficiently targets glioblastoma tumor-initiating cells leading to decreased proliferation and tumor growth.. Clin Cancer Res.

[34] Shay JW (1997). Telomerase in human development and cancer.. Journal of cellular physiology.

[35] Baird DM, Rowson J, Wynford-Thomas D, Kipling D (2003). Extensive allelic variation and ultrashort telomeres in senescent human cells.. Nat Genet.

[36] Choi J, Southworth LK, Sarin KY, Venteicher AS, Ma W (2008). TERT promotes epithelial proliferation through transcriptional control of a Myc- and Wnt-related developmental program.. PLoS genetics.

[37] Yu J, Hu K, Smuga-Otto K, Tian S, Stewart R (2009). Human induced pluripotent stem cells free of vector and transgene sequences.. Science.

[38] Yu J, Vodyanik MA, Smuga-Otto K, Antosiewicz-Bourget J, Frane JL (2007). Induced pluripotent stem cell lines derived from human somatic cells.. Science.

[39] Ebert AD, Yu J, Rose FF, Mattis VB, Lorson CL (2009). Induced pluripotent stem cells from a spinal muscular atrophy patient.. Nature.

[40] Zhao C, Blum J, Chen A, Kwon HY, Jung SH (2007). Loss of beta-catenin impairs the renewal of normal and CML stem cells in vivo.. Cancer Cell.

[41] Spisek R, Kukreja A, Chen LC, Matthews P, Mazumder A (2007). Frequent and specific immunity to the embryonal stem cell-associated antigen SOX2 in patients with monoclonal gammopathy.. J Exp Med.

[42] Duncan AW, Rattis FM, DiMascio LN, Congdon KL, Pazianos G (2005). Integration of Notch and Wnt signaling in hematopoietic stem cell maintenance.. Nat Immunol.

[43] Lessard J, Sauvageau G (2003). Bmi-1 determines the proliferative capacity of normal and leukaemic stem cells.. Nature.

[44] Reya T, Morrison SJ, Clarke MF, Weissman IL (2001). Stem cells, cancer, and cancer stem cells.. Nature.

[45] Masutomi K, Yu EY, Khurts S, Ben-Porath I, Currier JL (2003). Telomerase maintains telomere structure in normal human cells.. Cell.

[46] Wright WE, Piatyszek MA, Rainey WE, Byrd W, Shay JW (1996). Telomerase activity in human germline and embryonic tissues and cells.. Developmental genetics.

[47] Flores I, Canela A, Vera E, Tejera A, Cotsarelis G (2008). The longest telomeres: a general signature of adult stem cell compartments.. Genes & development.

[48] Nakamura TM, Morin GB, Chapman KB, Weinrich SL, Andrews WH (1997). Telomerase catalytic subunit homologs from fission yeast and human.. Science.

[49] Vulliamy T, Marrone A, Dokal I, Mason PJ (2002). Association between aplastic anaemia and mutations in telomerase RNA.. Lancet.

[50] Yamaguchi H, Calado RT, Ly H, Kajigaya S, Baerlocher GM (2005). Mutations in TERT, the gene for telomerase reverse transcriptase, in aplastic anemia.. N Engl J Med.

[51] Yuan X, Ishibashi S, Hatakeyama S, Saito M, Nakayama J (1999). Presence of telomeric G-strand tails in the telomerase catalytic subunit TERT knockout mice.. Genes Cells.

[52] Blasco MA, Lee HW, Hande MP, Samper E, Lansdorp PM (1997). Telomere shortening and tumor formation by mouse cells lacking telomerase RNA.. Cell.

[53] Akiyama M, Hideshima T, Shammas MA, Hayashi T, Hamasaki M (2003). Effects of oligonucleotide N3′–>P5′ thio-phosphoramidate (GRN163) targeting telomerase RNA in human multiple myeloma cells.. Cancer Res.

[54] Jackson SR, Zhu CH, Paulson V, Watkins L, Dikmen ZG (2007). Antiadhesive effects of GRN163L–an oligonucleotide N3′->P5′ thio-phosphoramidate targeting telomerase.. Cancer research.

[55] Rossi A, Russo G, Puca A, La Montagna R, Caputo M (2009). The antiretroviral nucleoside analogue Abacavir reduces cell growth and promotes differentiation of human medulloblastoma cells.. Int J Cancer.

[56] Bagheri S, Nosrati M, Li S, Fong S, Torabian S (2006). Genes and pathways downstream of telomerase in melanoma metastasis.. Proc Natl Acad Sci U S A.

[57] Hao LY, Armanios M, Strong MA, Karim B, Feldser DM (2005). Short telomeres, even in the presence of telomerase, limit tissue renewal capacity.. Cell.

[58] Sarin KY, Cheung P, Gilison D, Lee E, Tennen RI (2005). Conditional telomerase induction causes proliferation of hair follicle stem cells.. Nature.

[59] Li S, Crothers J, Haqq CM, Blackburn EH (2005). Cellular and gene expression responses involved in the rapid growth inhibition of human cancer cells by RNA interference-mediated depletion of telomerase RNA.. J Biol Chem.

[60] Samper E, Fernandez P, Eguia R, Martin-Rivera L, Bernad A (2002). Long-term repopulating ability of telomerase-deficient murine hematopoietic stem cells.. Blood.

[61] Allsopp RC, Morin GB, DePinho R, Harley CB, Weissman IL (2003). Telomerase is required to slow telomere shortening and extend replicative lifespan of HSCs during serial transplantation.. Blood.

[62] Guzman ML, Rossi RM, Karnischky L, Li X, Peterson DR (2005). The sesquiterpene lactone parthenolide induces apoptosis of human acute myelogenous leukemia stem and progenitor cells.. Blood.

[63] Park JI, Venteicher AS, Hong JY, Choi J, Jun S (2009). Telomerase modulates Wnt signalling by association with target gene chromatin.. Nature.

[64] Greipp PR, San Miguel J, Durie BG, Crowley JJ, Barlogie B (2005). International staging system for multiple myeloma.. J Clin Oncol.

